# *Talaromyces marneffei* and *Mycobacterium tuberculosis* co-infection in a patient with high titer anti-interferon-γ autoantibodies: a case report

**DOI:** 10.1186/s12879-021-07015-5

**Published:** 2022-01-28

**Authors:** Ye Qiu, Mianluan Pan, Zhenming Yang, Wen Zeng, Hui Zhang, Zhengtu Li, Jianquan Zhang

**Affiliations:** 1 Department of Respiratory and Critical Medicine, The Eighth Affiliated Hospital, Sun Yat-Sen University, Shenzhen, 518000 Guangdong China; 2 Department of Comprehensive Internal Medicine, The Affiliated Tumor Hospital of Guangxi Medical University, Nanning, 530021 Guangxi China; 3 Department of Respiratory and Critical Medicine, The First Affiliated Hospital of Guangxi Medical University, Nanning, 530021 Guangxi China; 4 State Key Laboratory of Respiratory Disease, National Clinical Research Center for Respiratory Disease, Guangzhou Institute of Respiratory Health, The First Affiliated Hospital of Guangzhou Medical University, Guangzhou, 510120 China

**Keywords:** *Talaromyces marneffei*, *Mycobacterium tuberculosis*, Anti-interferon-gamma autoantibody, HIV-negative patient, Case report

## Abstract

**Background:**

High-titer anti-interferon (IFN)-γ autoantibodies are strongly associated with intracellular pathogens such as nontuberculous mycobacteria and *Talaromyces marneffei*, but they are not as commonly associated with *Talaromyces marneffei* co-infected with *Mycobacterium tuberculosis*.

**Case presentation:**

Herein, we report a case of an HIV-negative Chinese man with a severe, disseminated co-infection of *Talaromyces marneffei* and *Mycobacterium tuberculosis*, who had a high-titer of anti IFN-γ autoantibodies and a CFI heterozygous nonsense gene mutation. The patient rapidly developed sepsis and died. Through by flow cytometry for CD4^+^ T cells’ intracellular phosphorylated STAT-1 and Th1 cells (CD4^+^ IFN-γ^+^ cells), we found that the patient’s serum can inhibited IFN γ-induced CD4^+^ T cells’ STAT-1 phosphorylation and Th1 cell differentiation in normal peripheral blood mononuclear cells, but this phenomenon was not observed in normal control’s serum. In addition, the higher serum concentration in the culture medium, the more obvious inhibition of Th1 cell differentiation.

**Conclusions:**

For HIV-negative individuals with relapsing, refractory, fatal double or multiple intracellular pathogen infections, especially *Talaromyces marneffei*, clinicians should be aware that if they might be dealing with adult-onset immunodeficiency syndrome due to high-titer anti-IFN-γ autoantibodies. Systematic genetic and immunological investigations should also be performed.

**Supplementary Information:**

The online version contains supplementary material available at 10.1186/s12879-021-07015-5.

## Background

Adult-onset immunodeficiency syndrome due to high-titer anti-interferon (IFN)-γ autoantibodies is considered to be a susceptibility factor for intracellular pathogens infection, especially nontuberculous mycobacteria and *Talaromyces marneffei* in Southeast Asia [1–3]. However, the specific mechanism of immune deficiency of anti-IFN-γ autoantibodies is still unclear. In addition, anti-IFN-γ autoantibodies have not widely been cognizant in tuberculosis [4]. Thus, we report a case of a 56-year-old HIV-negative Chinese man, with a high levels of anti-IFN-γ autoantibodies, simultaneously diagnosed disseminated *Mycobacterium tuberculosis* and *Talaromyces marneffei* co-infection by using metagenomics next-generation sequencing (mNGS), aims to attract clinical attention and attempts to study the possible immune deficiency mechanism of anti-IFN-γ autoantibodies.

## Case presentation

**Fig. 1:**
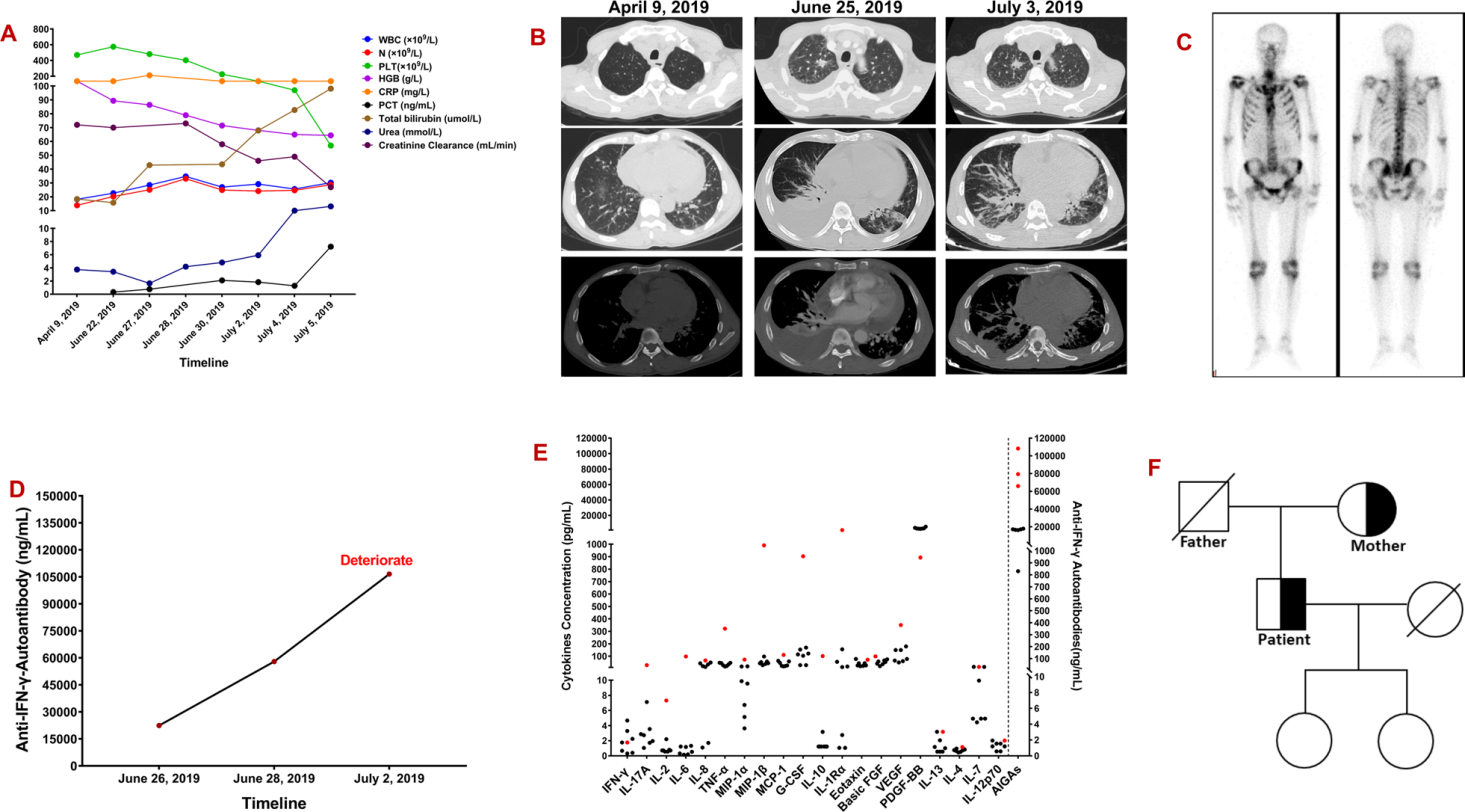
**A** Inflammatory markers, liver, and kidney function: white blood cell (WBC) and neutrophil (N) counts, and C-reactive protein (CRP), procalcitonin (PCT), urea and total bilirubin levels rapidly increased; platelets (PLT), hemoglobin (HGB) level, and creatinine clearance decreased rapidly. **B** Computed tomography dynamic monitoring series: pulmonary lesions, pleural effusions, pericardial effusions and osteolysis. **C** Emission computed tomography: significantly increased uptake in multiple ribs and vertebrae, left sacroiliac spine, and left acetabulum. **D** The patient’s anti-IFN-γ autoantibody titer increased significantly as the condition worsened during the disease course. **E** Multiplex screening of serum from the patient and 7 normal control plasmas for cytokines and anti-IFN-γ autoantibodies. **F** Pedigree tree. Whole-genome sequencing of the proband (patient) and his mother revealed a heterozygous nonsense mutation (c.559 C>T; p. Arg187*) in CFI

A 56-year-old Chinese man with coronary atherosclerotic heart disease was admitted to the local hospital on June 22, 2019 for a 4-month period due to expectoration, fever (body temperature: 39–40 °C), weight loss, and multiple lymphadenopathy. The patient also had significantly increased white blood cell (WBC) and neutrophil (N) counts as well as an increased erythrocyte sedimentation rate (ESR) and C-reactive protein (CRP) and procalcitonin (PCT) levels (Fig. 1A). He was nonresponsive to intermittent antibacterial therapy for 2 months (comprising cefoperazone sulbactam and moxifloxacin), and his condition deteriorated due to neck, armpit, and groin lymphadenopathy, jaundice, and proteinuria. Lymphocyte subset counts and percentages were normal. The patient had low levels of circulating factor I at 43% (normal value: 70–120%), but normal complement C3 and C4 levels. Thrombocytopenia, jaundice, and acute renal failure rapidly manifested later (Fig. 1A). HIV, antinuclear antibody, and anti-double stranded DNA tests were all negative. Chest computerized tomography (CT) revealed bilateral pulmonary infiltration with mediastinal lymphadenopathy, multiple bone destruction, and pleural and pericardial effusions (Fig. 1B). The emission CT showed a significantly increased uptake in multiple bones (Fig. 1C). A yellowish pleural effusion and its exudative manifestations and a marked increase in protein content and predominantly high levels of neutrophils (85%) were observed. The concentration of pleural fluid adenosine deaminase was 3.4 U/L. Histopathology of the lymph node and pulmonary lesions revealed granulomatous inflammation. Furthermore, no evidence of organisms or malignancy was identified in the bronchoscopy alveolar lavage fluid (BALF), blood, pleural effusion, bone marrow, lymphatic, or lung tissues when tested using microbial smears (negative acid-fast bacilli), cultures, and pathological examinations. However, *Mycobacterium tuberculosis* (TB) and *Talaromyces marneffei* were identified using Metagenomic next-generation sequencing (mNGS) [5] from the bronchoscopy analysis of the alveolar lavage fluid and cervical lymph nodes. The sputum and bone marrow were also analyzed for pathogen cultures using mNGS. Following a 3-day regimen of anti-tuberculosis treatment and antifungal therapy (amphotericin B liposome combined with voriconazole), the patient died of septic shock. On the 3rd day after his death, *Talaromyces marneffei* was isolated from the sputum and *Mycobacterium tuberculosis* was identified in the bone marrow using mNGS.

The patient, his mother, his two healthy daughters, and seven healthy controls, were recruited from the First Affiliated Hospital of Guangxi Medical University between June 2019 and July 2019. All subjects provided written informed consent. This study was approved by the Ethical Review Committee of the First Affiliated Hospital of Guangxi Medical University (2020.KY-E-032). Detailed methods for Anti-IFN-γ autoantibody assay, Bio-Plex™ 25 cytokine assay, flow cytometry, DNA extraction, DNA libraries and sequencing, and bioinformatics analysis are provided in Additional file 1.

**Fig. 2:**
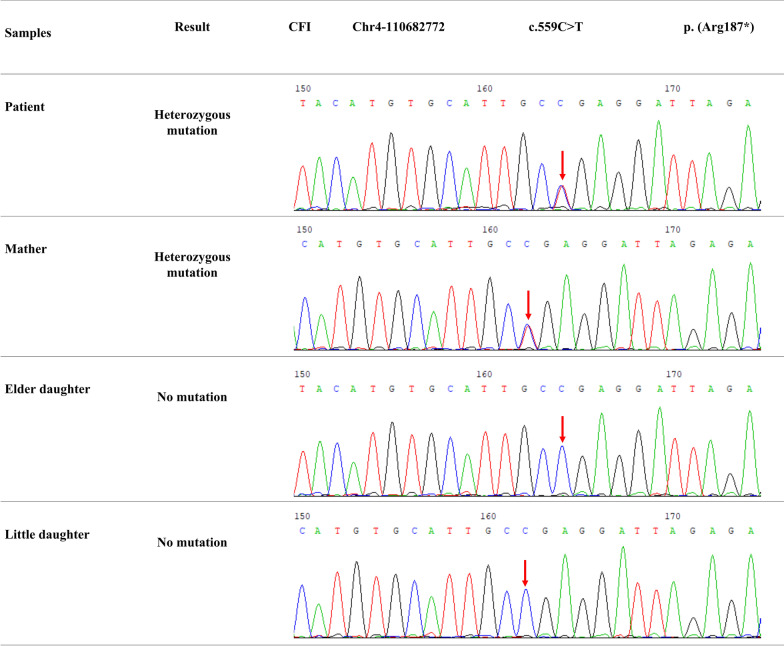
Whole-exome sequencing indicate a CFI heterozygous nonsense gene mutation (c.559 C>T; p. Arg187*) found in the patient and his mother

The patient’s serum showed a remarkably high-titer of anti-IFN-γ autoantibody, and the titer increased significantly as the condition worsened during the course of the disease. The patient’s serum IL-17 A, IL-2, IL-6, IL-8, TNF-α, MIP-1α, MIP-1β, MCP-1, G-CSF, IL-10, and IL-1Rα levels were also increased significantly than the seven healthy controls’ (Fig. 1D). However, the IFN-γ levels were in the same range as those in the healthy controls. Whole exome sequencing (WES) of the proband and his mother revealed a heterozygous CFI nonsense mutation (c.559 C>T; p. Arg187*; Figs. 1F and 2). The CFI gene (CFI, OMIM*217030) comprises 13 exons localized on chromosome 4q25 and its deficiency is inherited in an autosomal recessive manner. WES verification revealed that the patient’s mother was also a carrier of the heterozygous variant.

**Fig. 3:**
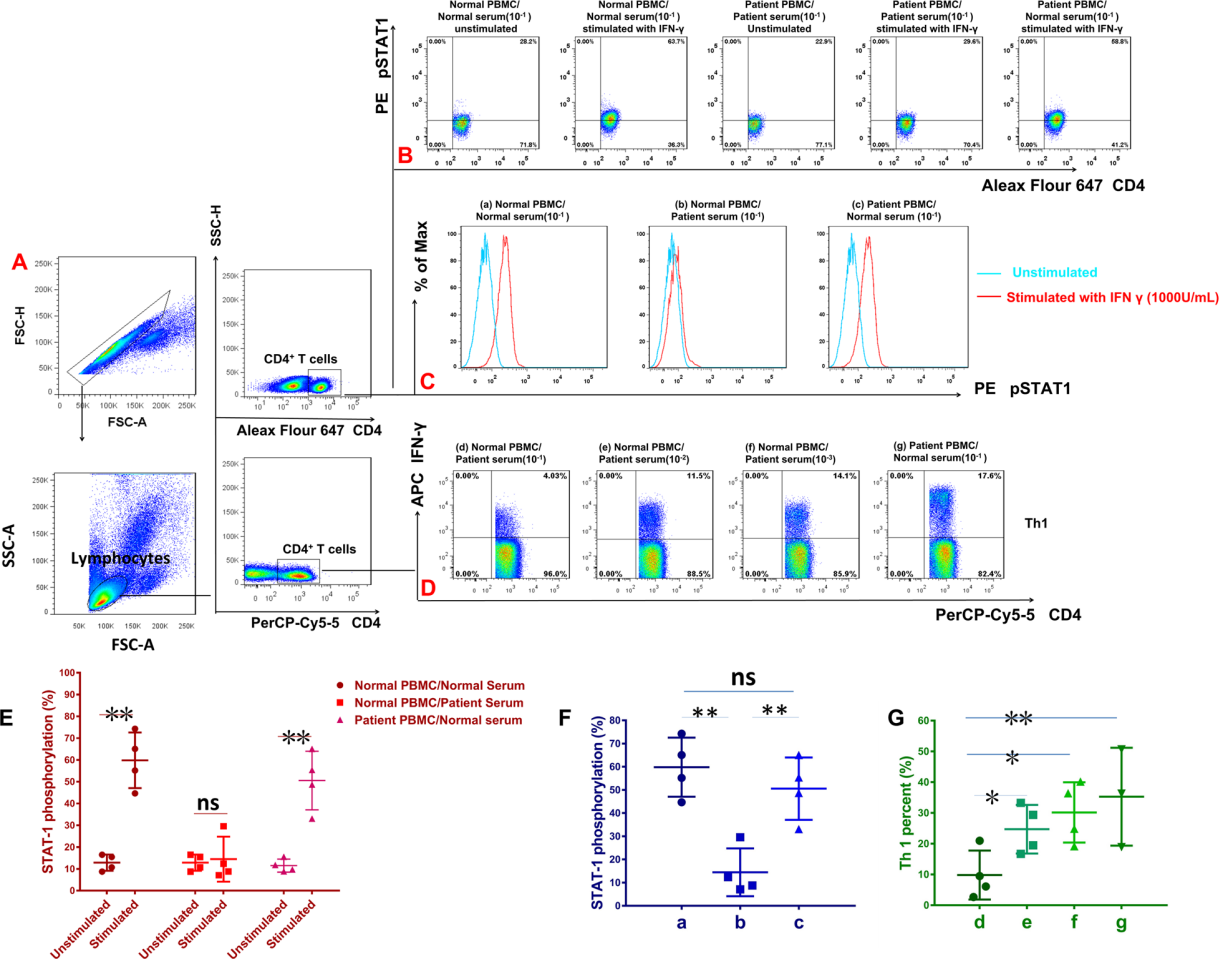
The expression of IFN-γ^+^ CD4^+^ T (Th1), and pSTAT-1^+^CD4^+^ T (CD4^+^ T cells intracellular phosphorylated STAT-1) cells in peripheral blood by Flow cytometry (n = 4). **A** Lymphocytes were identified based on their characteristic properties shown in by FSC and SSC. CD4^+^ T cells were gated from lymphocytes and total numbers of CD4+T cells were more than 25,000. **B** The dot plots of CD4^+^ T pSTAT-1 staining (CD4^+^ T cells intracellular phosphorylated STAT-1) cells in peripheral blood before and after stimulated with 1000 U/mL IFN γ and non-stimulated condition. **C** The histogram of CD4^+^ T pSTAT-1 staining (CD4^+^ T cells intracellular phosphorylated STAT-1) cells in peripheral blood before and after stimulated with 1000 U/mL IFN γ and non-stimulated condition. **D** The representative flow cytometric dot plots of IFN-γ^+^ CD4^+^ T cells (Th1). **E** The CD4^+^ T cells intracellular phosphorylated STAT-1 under stimulated with 1000 U/mL IFN γ and non-stimulated in three condition. **F** The CD4^+^ T cells intracellular phosphorylated STAT-1 under stimulated with 1000 U/mL IFN γ. **G** Th1 cells in the normal PBMCs and patients PBMCs co-culture with patient or normal serum. The patient’s serum but not the control serum inhibited IFN γ-induced CD4^+^ T cells STAT-1 phosphorylation in the normal peripheral blood mononuclear cells (PBMCs), whereas when the patient’s PBMCs were washed free of autologous serum they demonstrated normal IFN γ-induced STAT-1 phosphorylation. The patient but not control serum inhibited Th1 cell differentiation in the normal PBMCs. Data are expressed as median with interquartile range. n = 4 for each group. Statistical comparisons were made using the Kruskal–Wallis test followed by Bonferroni test. *P < 0.05. **P < 0.01. *FSC* forward scatter, *SSC* side scatter

Through by flow cytometry for CD4^+^ T cells’ intracellular phosphorylated STAT-1 and Th1 cells (CD4^+^ IFN-γ^+^ cells), we found that the patient’s serum can remarkably inhibited IFN γ-induced CD4^+^ T cells’ STAT-1 phosphorylation and Th1 cell differentiation in normal peripheral blood mononuclear cells, but this phenomenon was not observed in normal control’s serum. However, when the patient PBMCs were washed free of the patient’s serum, they demonstrated normal IFN γ-induced STAT-1 phosphorylation in CD4^+^ T cells and Th1 cell differentiation (Fig. 3). In addition, the higher serum concentration in the culture medium, the more obvious inhibition of Th1 cell differentiation.

## Discussion and conclusion

A high-titer of anti-IFN-γ autoantibodies is strongly associated with intracellular pathogens especially in *Nontuberculous mycobacteria* and *Talaromyces marneffei* [1]. Cao et al. found that there was a high prevalence of neutralizing anti-IFN-γ autoantibodies (94.8%) in 58 HIV-negative adults with severe *Talaromyces marneffei* infections who were otherwise healthy [5]. In our previously researches, we found that it showed that adult-onset immunodeficiency syndrome, due to high-titer of anti-IFN-γ autoantibodies were the most common underlying immunodeficiency in HIV-negative *Talaromyces marneffei* infection patients [6]. In addition, the ratio of actual value to cut-off value of anti-IFN-γ autoantibodies was an independent risk factor of TM infection [2]. However, the immune deficiency mechanism of anti-IFN-γ autoantibodies in *Talaromyces marneffei* infection is less clear.

Lightly increased levels of anti-IFN-γ autoantibodies were found in patients with severe pulmonary tuberculosis [1]. However, the neutralization or biological activity of lightly increased levels of anti-IFN-γ autoantibodies in these pulmonary tuberculosis were not proved [7]. Meanwhile, remarkable high titer of anti-IFN-γ autoantibodies found in tuberculosis is rare reported. Before our case, there was only one Thai woman with a disseminated tuberculosis single infection and a dramatic paradoxical inflammatory response after treatment initiation, was found to have a high-titer of neutralizing anti-IFN-γ autoantibodies [4]. However, our patient had disseminated tuberculosis and a TM co-infection combined with a high level of anti-IFN-γ autoantibodies, phenomena that triggered an inflammatory storm, resulting in rapid death. Moreover, our patient’s serum could inhibit IFN γ-induced CD4^+^ T cells’ STAT-1 phosphorylation and Th1 cell differentiation in normal PBMCs. These observations suggest that the immunodeficiency mechanism of the anti-IFN-γ autoantibody may include inhibition of the CD4^+^ T cells’ IFN-γ/pSTAT-1/Th1 pathway, ultimately leading to a severely compromised Th1 response. Thus, Th1 cells immunodeficiency due to anti-IFN-γ autoantibodies was the cause of the severe and fatal multiple intracellular pathogen infections in this HIV-negative adult. Furthermore, the levels of proinflammatory cytokines were increased significantly in our patient, especially IL-17 A, IL-6, IL-8, TNF-α, G-CSF, and IL-10.

Browne, et al. identified a major epitope using anti-interferon-γ autoantibodies in patients with mycobacterial disease that showed a homology to the *Aspergillus* protein and found that anti-IFN-γ autoantibodies cross-reacted with *Aspergillus* and *Mycobacterium intracellulare* Noc2 [8]. These results suggest that when a patient is infected with TM and TB, these forms of molecular mimicry may trigger the production of these autoantibodies. Thus, the anti-IFN-γ autoantibody titer increased significantly as the condition worsened during the disease course.

In conclusion, clinicians should be aware that HIV-negative individuals with relapsing, refractory, fatal multiple intracellular pathogen infections, may have multiple immunodeficiencies, including primary immunodeficiency and/or secondary immunodeficiency, that are unrecognized. Thus, systematic genetic testing and immunological investigations should be performed for such patients.

## Supplementary Information

**Additional file 1.** Detailed methods for Anti-IFN-γ autoantibody assay, Bio-Plex™ 25 cytokine assay, flow cytometry, DNA extraction, DNA libraries and sequencing, and bioinformatics analysis are provided in Additional file.

## Data Availability

The DNA sequencing of the patient’s data were submitted to the NCBI GenBank database (The GenBank accession number for the patient’s nucleotide sequence: OL537177). The other data generated or analyzed during this study are included in this published article.

## Abbreviations

IFN-γ: Interferon-γ
PBMCs: Peripheral blood mononuclear cells
mNGS: Metagenomics next-generation sequencing
WBC: White blood cell
N: Neutrophil
ESR: Erythrocyte sedimentation rate
CRP: C-reactive protein
PCT: Procalcitonin
CT: Computerized tomography
BALF: Bronchoscopy alveolar lavage fluid
EDTA: Ethylene diamine tetraacetic acid
PBS: Phosphate buffer saline
ELISA: Enzyme-linked immunosorbent assay
MFI: Mean fluorescent intensity
PMA: Phorbol myristate acetate
DNB: DNA Nanoballs
PMDB: Pathogens Metagenomics Database
WES: Whole exome sequencing
