# Supplementary material for: Talaromyces marneffei and Mycobacterium tuberculosis co-infection in a patient with high titer anti-interferon-γ autoantibodies: a case report
Source: BMC Infect Dis. 2022 Jan 28;22:98. doi: 10.1186/s12879-021-07015-5 (PMC8796477; doi:10.1186/s12879-021-07015-5)
Supplement: Supplementary file 1 — Additional file 1. Detailed methods for Anti-IFN-γ autoantibody assay, Bio-Plex™ 25 cytokine assay, flow cytometry, DNA extraction, DNA libraries and sequencing, and bioinformatics analysis are provided in Additional file. [file 12879_2021_7015_MOESM1_ESM.docx]

**Materials & Methods**

The patient, his mother, his two healthy daughters, and seven healthy controls, were recruited from the First Affiliated Hospital of Guangxi Medical University between June 2019 and July 2019. All subjects provided written informed consent. This study was approved by the Ethical Review Committee of the First Affiliated Hospital of Guangxi Medical University (2020.KY-E-032).

Whole blood samples from the subjects were collected in ethylene diamine tetra acetic acid (EDTA)-treated tubes. Peripheral blood mononuclear cells (PBMCs) were separated using the Lymph- prep (Stemcell Technologies, Canada) centrifugation method. Briefly, fresh blood samples were mixed with an identical volume of phosphate buffer saline (PBS) and were carefully placed on the surface of the Lympho-prep separation medium. After centrifugation at 500 × *g* for 20 min at 28 °C, PBMCs were collected at the interphase and washed with PBS by centrifugation for 10 min at 300 × *g* [1]. Serum samples, obtained under sterile conditions, were retrieved from a serum bank maintained by our laboratory (stored at −80 °C).

***Anti-IFN-γ autoantibody assay***

All samples were tested after their first thaw from the -80 ℃storage. Anti-IFN-γ autoantibody titer in the serum of the patient, his family members, and the 7 healthy volunteers was determined by enzyme-linked immunosorbent assay kit. Serum anti-IFN-γ autoantibody levels were determined using an enzyme-linked immunosorbent assay kit (ELISA; USCN Life Science Inc., Wuhan, China), as per the manufacturer’s protocol [2]. The normal range for the anti-IFN-γ autoantibody concentration was defined by the 99th percentile for the healthy controls and was estimated using the log-normal distribution. Outlying concentrations were classified as positive for anti-IFN-γ autoantibody [3].

***28 Cytokine assay***

A Bio-Plex^™^ cytokine assay (Bio-Rad) was used to measure the cytokine levels in tear samples, and the analyses were performed in triplicate. Twenty-five cytokines, including MIP-1β, IL-6, IFN- γ, IL-1Ra, IL-5, GM-CSF, TNF-α, RANTES, IL-2, IL-1β, exotoxin, basic FGF, VEGF, PDGF-BB, IP-10, IL-13, IL-4, MIP-1α, IL-10, G-CSF, IL-15, IL-7, IL-12p70, IL-17A, and IL-9, were assessed [4]. The concentrations were calculated based on the mean fluorescent intensity (MFI) using Luminex200 IS V2.1. Standard curves were generated from the reference cytokine gradient concentrations; the concentrations of these cytokines in the tear samples were calculated based on the standard curves. Three cytokines (IL-21, IL-22, and IL-23) were further detected using ELISA (R&D Systems, Minneapolis, MN, USA). Analyses were performed as per the manufacturer’s instructions for each ELISA kit, and in triplicate.

***Flow cytometry for Th1 cells (identified as CD4^+^ IFN-γ^+^ cells)***

Fresh PBMCs were isolated from the blood of the patient and four healthy donors using the Lymphoprep (Stemcell Technologies) centrifugation method. Serum from the patient (July 2, 2019) and donors was serially diluted (10^−1^ to 10^−3^). The PBMCs (1.0 × 10^6^ cells/well) were then stimulated in duplicate with 50 ng/mL phorbol myristate acetate (PMA) and 1 µg/mL ionomycin (both obtained from Sigma, St. Louis, MO, USA) in RPMI 1640 media in the presence of GolgiStop at 37 °C in an atmosphere of 5% CO_2_ for 5 h, with or without patient serum, and using different dilution gradients (1:10, 1:100, 1:1000). After stimulation, the cells were surface stained with an anti-CD4 monoclonal antibody (Percp-cy5.5; BD Pharmingen) for 30 min at 4 °C and then fixed and permeabilized for 20 min at 4 °C using a Fixation/Permeabilization Solution (BD Pharmingen). Subsequently, the cells were washed with Perm/Wash buffer (BD Pharmingen), and then incubated with an anti-IFN-γ monoclonal antibody (APC; BD Pharmingen) at 4 °C for 30 min. Lymphocytes were identified based on their characteristic FSC and SSC. Th1 cells were identified as CD4^+^ IFN-γ^+^ cells [1].

***Flow cytometry for CD4 ^+^ T cells’ intracellular phosphorylated STAT-1***

Fresh PBMCs were isolated from the blood of the patient and four healthy donors using the Lymphoprep (Stemcell Technologies) centrifugation method. Serum from patients (July 2, 2019) and donors was serially diluted. Then, PBMCs (1.0×10^6^ cells/well) were cultured in the presence of normal or patient serum with RPMI 1640 media at 37 °C in an atmosphere of 5% CO_2_, and then unstimulated or stimulated for 30 min with or without IFN-γ (1000 U/mL). The cells were fixed and stained for determining the CD4^+^T cells intracellular phosphorylated STAT-1 levels using flow cytometry [5]. Lymphocytes were identified based on their characteristic FSC and SSC. Intranuclear staining was performed using an anti-CD4 monoclonal antibody (Alexa Flour 647; BD Pharmingen) and anti–phospho–signal transducer and activator of transcription 1 (STAT1; PE; BD Pharmingen). CD4^+^T cells STAT-1 phosphorylation was determined using CD4^+^ pSTAT-1^+^ [6].

***Sample processing and DNA extraction***

Samples from the patient (1.5–3 mL) were collected using standard procedures. A 1.5 mL microcentrifuge tube containing a 0.6 mL sample and 250 μL of 0.5 mm glass beads was attached to a horizontal platform on a vortex mixer and agitated vigorously at 2800–3200 rpm for 30 min. Then 7.2 μL lysozyme was added to induce a cell wall-breaking reaction; 0.3 mL of this sample was aliquoted into a new 1.5 mL microcentrifuge tube, and DNA was extracted using the TIANamp Micro DNA Kit (DP316, TIANGEN BIOTECH), according to the manufacturer’s recommendations [7].

***Construction of DNA libraries and sequencing***

DNA libraries were constructed using DNA-fragmentation, end-repair, adapter-ligation, and PCR amplification. Agilent 2100 was used for the quality control of the DNA libraries. Qualified libraries were pooled and DNA Nanoballs (DNB) were prepared and sequenced using the BGISEQ-50 /MGISEQ-2000 platform [8].

***Bioinformatic analysis***

High-quality sequencing data were generated by removing low-quality reads, followed by computational subtraction of the human host sequences mapped to the human reference genome (hg19) using Burrows-Wheeler Alignment. The low-complexity reads in the remaining data were classified by simultaneously aligning them to the Pathogens Metagenomics Database (PMDB), consisting of bacteria, fungi, viruses, and parasites. The classification reference databases were downloaded from NCBI (ftp://ftp.ncbi.nlm.nih.gov/genomes/). RefSeq contains 4,945 whole genome sequences of viral taxa, 6,350 bacterial genomes or scaffolds, 1,064 fungi related to human infection, and 234 parasites associated with human diseases [9].

**References**

1. Zhang H, Qiu SL, Tang QY, et al. Erythromycin suppresses neutrophil extracellular traps in smoking-related chronic pulmonary inflammation. Cell Death Dis. 2019;10(9):678.
2. Qiu Y, Huang J, Li Y, et al. Talaromyces marneffei and nontuberculous mycobacteria co-infection in HIV-negative patients. Sci Rep. 2021;11(1):16177.
3. Browne SK, Burbelo PD, Chetchotisakd P, et al. Adult-onset immunodeficiency in Thailand and Taiwan. N Engl J Med **2012**; 367(8): 725-34.
4. Bateman RM, Sharpe MD, Jagger JE, et al. 36th International Symposium on Intensive Care and Emergency Medicine: Brussels, Belgium. Crit Care. 2016;20(Suppl 2):94.
5. Li HR, Cai SX, Chen YS, et al. Comparison of Talaromyces marneffei Infection in Human Immunodeficiency Virus-positive and Human Immunodeficiency Virus-negative Patients from Fujian, China. Chin Med J (Engl). 2016;129(9):1059-6.
6. Kang YH, Biswas A, Field M, Snapper SB. STAT1 signaling shields T cells from NK cell-mediated cytotoxicity. Nat Commun. 2019;10(1):912.
7. Long Y, Zhang Y, Gong Y, et al. Diagnosis of Sepsis with Cell-free DNA by Next-Generation Sequencing Technology in ICU Patients. Arch Med Res. 2016;47(5):365-371.
8. Jeon YJ, Zhou Y, Li Y, et al. The feasibility study of non-invasive fetal trisomy 18 and 21 detection with semiconductor sequencing platform. PLoS One. 2014;9(10):e110240.
9. Li H, Durbin R. Fast and accurate long-read alignment with Burrows-Wheeler transform. Bioinformatics. 2010;26(5):589-95.
